# Soil water use efficiency under integrated soil management practices in the drylands of Kenya

**DOI:** 10.1016/j.heliyon.2023.e16145

**Published:** 2023-05-15

**Authors:** O. Nathan Oduor, Monicah Mucheru-Muna, Nyanuga Jayne Mugwe, Isaya Sijali, George Nyabuga, N. Daniel Mugendi

**Affiliations:** a Department of Water and Agricultural Resource Management, University of Embu, P.O. Box 6-60100, Embu, Kenya; b Department of Environmental Sciences, Kenyatta University, P.O. Box 43844-00100, Nairobi, Kenya; c Department of Agricultural Sciences and Technology, Kenyatta University, P.O. Box 43844-00100, Nairobi, Kenya; d Food Crops Research Centre-Kabete, Kenya Agricultural and Livestock Research Organization (KALRO), PO Box 14733-00800, Nairobi, Kenya; e Graduate School of Media and Communication, Aga Khan University, P.O. Box 30270-00100, Nairobi, Kenya

**Keywords:** Animal manure, Ferralsols, Mineral fertilizer, Minimum tillage with mulch, Soil moisture, Tied ridges

## Abstract

Soil moisture scarcity and soil fertility decline in the drylands contribute to declining crop productivity. The possible synergistic effects of integrating soil & water conservation, and soil fertility management practices on soil moisture, and hence water use efficiency (WUE) in the drylands of Tharaka-Nithi County in Kenya was assessed. The experiment was laid in a three by three split plot arrangement, with four replications, for four cropping seasons. Minimum tillage with mulch, tied ridges, and conventional tillage formed the main plot factors. The sub-plot factors included animal manure plus fertilizer at 120, 60, and 30 N kg ha^−1^. There was significant improvement in soil moisture by 35 and 28% by minimum tillage with mulch and tied ridges, respectively, compared to conventional tillage. Manure plus fertilizer rates of 120 and 60 N kg ha^−1^ had significantly lower soil moisture by 12 and 10%, respectively than the 30 N kg ha^−1^ across the seasons. The WUE was significantly enhanced by 150 and 65% under minimum tillage with mulch and tied ridges, respectively, compared to conventional tillage. Compared with 30 N kg ha^−1^, the 120 N kg ha^−1^ and 60 kg ha^−1^ significantly enhanced the WUE by 66 and 25%, respectively. Across the seasons, the best treatment combination for improving WUE was minimum tillage with mulch at 120 N kg ha^−1^ rate of manure plus fertilizer.

## 1 Introduction

Scarce and erratic rainfall patterns and declining soil fertility continue to ravage rainfall-dependent agriculture globally [[1], [2], [3]]. This is despite the expected surge of 60% in global food demand by 2050 compared to 2007, with the rise in sub-Saharan Africa (SSA) expected to be greater [4]. The rise is attributed to the constant population increase [4,5]. Rainfall scarcity and unreliability cause moisture stress to the crop, considering smallholder agriculture in SSA is majorly rain-fed, with over 95% of agricultural land under rainfed farming [6]. This has made agricultural productivity vulnerable and risky venture, especially in the drylands [1,3]. Soil nutrient mining without adequate amendment, erosion, and leaching losses contributes to declining soil fertility Poor agronomic practices among farmers that lack adaptive capacity exacerbate the decline in crop productivity [8].

Farmers constantly plough their lands, continuously mining the soil nutrients, without adequate addition of soil amendments [9]. Equally, farmers' practices lack soil moisture conservation mechanisms, and hence cannot alleviate soil moisture stress, especially in drought-prone areas like the Kenyan drylands [5,10]. Soil hydrological properties, which are critical for soil moisture conservation have been derailed as a result [2]. This has contributed to low water use efficiency (WUE) and reduced crop productivity. Efforts to ensure every drop of rainwater received is meticulously used for crop production are key if food productivity is to match the demand of the constantly growing population [11].

Minimum tillage (MT) improves soil water retention, enhances the infiltration rate of the soil, alleviates surface runoff losses, and enhances crop yield as well as the efficiency of nutrient utilization [11]. Minimum tillage reduces soil disturbance that encourages the continuity of the soil pores, hence contributing to improved soil infiltration and water retention [12]. However, minimum tillage has been faulted for exposing soil nutrients and organic amendments to erosion losses as they are normally concentrated on the soil surface under MT, and thus prone to surface runoff losses [7]. Whereas conventional tillage reduces such losses, it leads to soil disturbance, causing surface sealing and soil crusting, which lower the rate of infiltration, and hence increase runoff losses [13]. Nevertheless, minimum tillage with mulch combined with organic resources reduces runoff losses [14]. This is because the organic mulch adds to soil organic matter which enhances the aggregate stability of the soil, and thus water-holding capacity [2]. Conversely, there are contradicting reports on the performance of minimum tillage compared to conventional tillage, especially when integrated with other soil management approaches [15]. The effectiveness of various tillage methods, however, depends on the type of soil and the climate under consideration [16]. Therefore, further investigation on the impact of minimum and conventional tillage, when combined with other soil management strategies on agricultural productivity in tropical environments is necessary.

Tied ridging promotes water retention of overland water flow in ditches, resulting in reduced surface runoff loss [2]. Nevertheless, the tied ridging encouraged overtopping that enhanced soil erosion, especially under high rainfall events [17,18]. In addition, the effect of tied ridges on soil fertility and crop yield is not as well researched as on soil moisture conservation [19]. As a result, its efficacy on soil fertility and agricultural productivity should be investigated, particularly when combined with other soil and water conservation techniques over a range of rainfall regimes [20].

The combined use of mineral fertilizer with animal manure has the most sustainable gain in crop yield per unit of water used, besides being accessible within the socio-economic conditions of most farmers [21]. The synergy under the combined effect enhances nutrient release and eventual uptake by the crop, thus the high nutrient use efficiency [22]. Furthermore, the combined use improves soil infiltration rate, aggregation, bulk density, and hydraulic conductance, reduces crust strength, and efficient water utilization [13]. However, proper management is required for the realization of the desired effect. For example, under poor management, N immobilization occurs, especially, when organic resources with a high C: N ratio are combined with mineral fertilizer [3]. This causes an eventual reduction in crop yield as nitrogen meant for crop uptake is utilized by the soil microbes in the decomposition process [23]. Nevertheless, most studies have reported that combined organic and mineral fertilizers boosted crop yield [10,24,25]. The effectiveness of the integrated use of animal manure plus mineral fertilizer, combined with other soil management practices needs further evaluation.

Improved WUE not only requires soil moisture conservation but also improved soil fertility among other crop production factors, simultaneously [11]. Integrating practices with the ability to enhance soil fertility and conserve soil moisture is the most effective and sustainable approach toward enhancing soil WUE. The objective of the study was to assess the effect of integrating soil & water conservation, and soil fertility management practices on soil moisture, and hence WUE in the drylands of Kenya.

## 2 Materials and methods

### 2.1 Study area

**Fig. 1:**
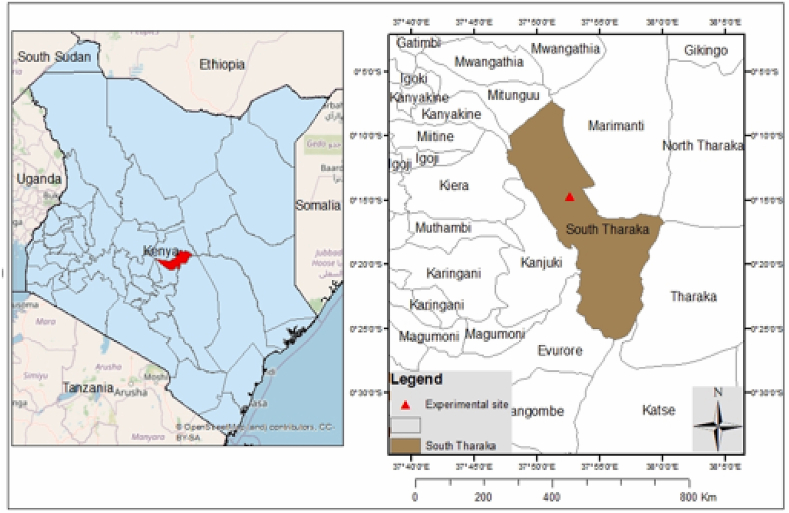
Map of the study area.

The experiment was carried out at Nkarini Secondary School in Tharaka-Nithi County (Fig. 1). The site is at latitude 0°14'43" S and longitude 37°52'37" E with an elevation of approximately 790 m above sea level. The annual mean temperature is around 28 °C with a mean annual rainfall of about 800 mm [26]. The region has a bimodal rainfall pattern with the Long Rains (LR) lasting from March to June, and Short Rains (SR) from October to January. The dominant crops grown are drought tolerant, including sorghum (*Sorghum bicolor* L.) and green grams (*Vigna radiate* L.). Dominant soil types are the ferralsols, a heavily eroded soil [26].

### 2.2 Field experimental design

A three by three split-plot experimental design with four replications was adopted. Minimum tillage with mulch, conventional tillage and tied ridges were the main plot factors. Manure plus fertilizer at 120, 60, and 30 N kg ha^−1^ were the subplot factors. Manure and fertilizer at 30 N kg ha^−1^ under conventional tillage were used as the control (Table 1). This is what the majority of the farmers practice in the region, where they apply inadequate mineral fertilizer rates below the recommendation, and constantly plough their land conventionally [9].

**Table 1:** Soil fertility and, soil & water conservation approaches tested in the experiment in the drylands of Tharaka-Nithi County, Kenya.

Soil fertility inputs	Soil and water conservation practices	Manure rate (N equivalent in Kg/ha)	Mineral fertilizer rate (Kg/ha)	
			N	P
Manure + Fertilizer (Low)	Minimum tillage with crop residue	15	15	78
	Tied ridging	15	15	78
	Conventional tillage	15	15	15
Manure + fertilizer (Moderate)	Minimum tillage with crop residue	30	30	96
	Tied ridging	30	30	96
	Conventional tillage	30	30	30
Manure + fertilizer (High)	Minimum tillage with crop residue	60	60	132
	Tied ridging	60	60	132
	Conventional tillage	60	60	60

N-Nitrogen; P-Phosphorous.

### 2.3 Field experimental management

The plots were 6.75 by 3 m in size (m). The plot spacing was 1 m, while the spacing between the blocks was 3 m. The *gadam* sorghum (*Sorghum bicolor* L.) variety was the test crop. Inter-row spacing for sorghum was 0.75 m, while the intra-row was 0.20 m. Three seeds were planted per planting hole and thinned to two plants a week after emergence. This ensured sorghum population density of 133,333 plants ha^−1^ was maintained. The land was tilled to 15 cm depth using a hand hoe under conventional tillage, with the same used for weeding. The land was surface cleared and weeding done by uprooting under minimum tillage. Two weeks before planting, animal manure was applied along the planting lines and mixed with the soil at 15 cm depth under minimum tillage. Animal manure was integrated into the entire plot at the same depth under conventional tillage. Manure was analyzed for nutritional content (N = 0.93%, P = 0.20%, and K = 2.47%) and the equivalence of 15 kg ha^−1^ was applied for low, 30 kg ha^−1^ for moderate and 60 kg ha^−1^ for high nitrogen and phosphorus rates.

Mineral fertilizer was applied at rates of 15, 30, and 60 N and P kg ha^−1^ for low, moderate, and high nitrogen and phosphorus rates, respectively. Phosphorus was further supplemented with Triple Super Phosphate (TSP) at the same rate of 60 P kg ha^−1^ across the plots. Crop residue mulch was applied at a rate of 5 Mg ha^−1^ on the minimum tillage plots two weeks after crop emergence. At the start of the season, tied ridges were constructed across the slope of the plots and repaired whenever they got damaged. The ridges were 0.15 m, whereas the ties were 0.10 m in height. The alternate distance between the ties was 1 m. The trial was set in April 2019 and lasted for four cropping seasons (LR 19, SR 19, LR 20 and LR 20).

### 2.4 Data collection

Throughout the study period, the meteorological parameters were monitored by the installed automated weather station at the site. The maximum and minimum temperatures and rainfall were measured daily. The rainfall data was used in the calculation of the start and end of the season, and the length of the crop growth period. The onset date was the first three days that received a cumulative rainfall of more than 40 mm in a season [27]. The cessation date was the end of the rains. The duration of the crop growing period was the difference between cessation and onset dates [1]. A dry spell within the season was established for every season. A dry spell was considered to be consecutive dry days of more than five sandwiched between wet days [28].

### 2.5 Runoff measurement

Each plot was fenced with corrugated iron sheets on the three sides. The iron sheets protruded 0.15 m above the soil surface. Fencing was to prevent lateral runoff overlap from one plot to the next or spillage of runoff from the plots. The slope gradient of the plots was approximately 2%. Runoff collection ditches are lined with plastic dam liners on the lower outlets of the plots for runoff collection. Runoff data were collected whenever a rainfall event generated runoff. The rainwater volume entering into runoff collection ditches directly was calculated and deducted from the total runoff amounts. The volume of runoff was measured by calibrated buckets to which the runoff was emptied.

### 2.6 Determination of crop yield

Sorghum grain yield was weighed at harvesting in every cropping season from the net plot area of 14.2 m^2^. Dickey-John MiniGAC® moisture meter was used in monitoring the grain moisture percentage while drying. Grain yield was then expressed to 12.5% moisture content after sun drying. The Stover sample was dried in an oven to constant weight, then used in the extrapolation of the total dry stover yield. Grain and stover yield was converted to a per hectare basis.

### 2.7 Soil moisture determination

Soil moisture was monitored daily from the planting time until harvesting for three seasons using time-domain reflectometry (TDR 200) meter equipment (Campbell Scientific, United States of America). The TDR sensors for soil moisture monitoring had not been installed at the start of the first cropping season due to technical challenges. Therefore, soil moisture was not monitored during the LR 19 season (first season). The moisture readings were taken from the vertically installed metallic probes at 0–20 and 20–40 cm depths. The TDR200 m was calibrated using the Topp et al. [29] equation;

(1) $${Ɵ}_{v}=-5.3\;x\;10^{-2}+2.92\;x\;10^{-2}\;K_{a}-5.5\;x10^{-4}K_{a}^{2}+4.3\;x\;10^{-6}\;xK_{a}^{3}$$

where ${Ɵ}_{v}$ is the volumetric soil water content and $K_{a}$ apparent permittivity

Soil moisture was then grouped into four crop growth stages for further processing. The crop growth stages included the vegetative growth stage, which was from the crop germination to the end of active vegetative development; the floral stage, which included flowering; the grain filling stage which include the soft dough and hard dough stages; and the physiological maturity stage.

### 2.8 Water use efficiency determination

The water balance equation [ [30]] was used in the calculation of the evapotranspiration (ET) using the ETo Calculator;

(2) ET=(P+I+C)−(R+D)−ΔSWS

(3) $$\Delta SWS=S_{initial}-S_{present}$$

where P is precipitation, I is irrigation, C is the upward flux, R is runoff loss, D is the drainage, ΔSWS (mm) is the difference in soil water storage at sowing ($S_{initial}$) and at harvesting ($S_{present}$).

For uniformity and standardization, it was assumed that underground upthrust (C), downward drainage (D), and surface cover were insignificant. There was no irrigation (I), as the experiment was rainfed. The final equation was;

(4) ET=P−R−ΔSWS

Water use efficiency was calculated from the yield and soil water relation for three seasons. The WUE was derived as per the method by Pereira et al. [31];

(5) $$WUE=\frac{B}{ET}$$

where WUE is water use efficiency, and B is either sorghum grain yield or total above-ground biomass (grain plus stover).

### 2.9 Limitation of the methodology

Underground upthrust (C) and downward drainage (D) were not measured despite the heavy rainfall in some seasons. However, treatment comparison was not influenced as they were all subjected to the same conditions.

### 2.10 Data analysis

The effect of treatment on soil moisture and WUE was analyzed by two-way analysis of variance using the general linear model in SAS software version 9.4 [32]. Tukey’s honestly significant difference test at p < 0.05 was used in the separation of means.

## 3 Results and discussion

### 3.1 Rainfall and temperatures during the experimental period

**Fig. 2:**
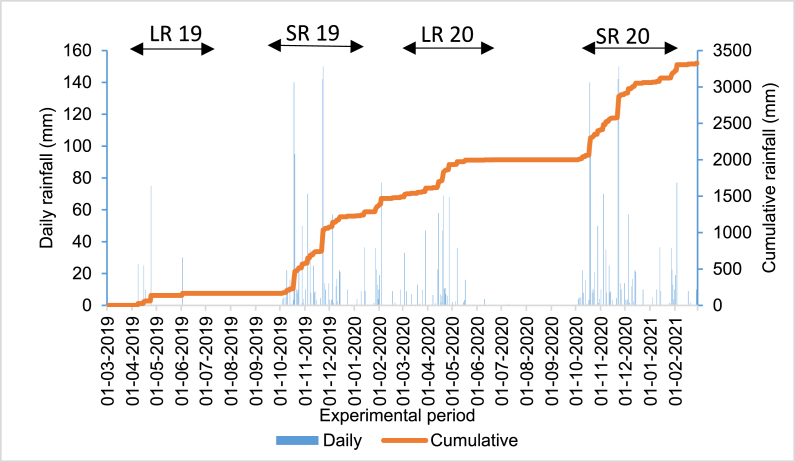
Rainfall pattern at the study site during the trial in the drylands of Tharaka-Nithi County, Kenya.

The rainfall amount received in the year 2019 was 1227 mm, while 1435 mm was received in 2020. The long rains (LR) 2019 and 2020 seasons received 140 and 400 mm, respectively. The short rains (SR) in 2019 and 2020 received 1242 mm and 695 mm, respectively (Fig. 2). During the LR19 season, a dry spell of 46 days was observed, while there was none in SR 19 season. The LR 20 season had 10 and 8-day dry spells, while the SR 19 season had a 6-day dry spell. Generally, there were more dry spells of greater magnitude during the long than the short rains. The duration of crop growth was 5, 116, 46, and 68 days during the LR19, SR 19, LR 20, and SR 20 seasons.

The temperature values ranged from 15 to 35 °C during the study period. Maximum temperatures were between 25 and 35 °C. Minimum temperatures ranged between 15 and 20 °C. There were no wide variations in temperature values across the seasons.

The high temperatures in the region set the threshold for a rainy day to be at 1 mm. This is because rainfall below this amount is likely to be evaporated back into the atmosphere [3]. Rainfall amounts received influenced the yield per season. From the study, crop yield was high during the season with high rainfall amounts (section 3.3). This is because soil moisture, which is solely from the rains, is among the major limiting factors to crop productivity [6]. Rainfall amount and distribution are key to crop development and performance [27]. The rainfall received affects the onset and cessation dates and the length of the crop growing season. Well distributed rainfall amounts across the season could result in high WUE even when the amount received was relatively low [1].

### 3.2 Treatment effect on soil moisture

Treatments had a significant influence on soil moisture during the study period at 0–40 cm soil depth at various sorghum growth stages (Table 2). Soil and water conservation practices influenced soil moisture. Minimum tillage with residue mulch improved soil moisture by 35–24%, whereas tied ridges improved soil moisture by 18–22% compared with conventional tillage. Manure plus fertilizer at 120 N kg ha^−1^ and 60 N kg ha^−1^ had lower soil moisture by 9–12% and 10-8% compared with the 30 N kg ha^−1^ across the seasons and crop growth stages. The combination of minimum tillage with mulch with manure plus fertilizer at 30 kg ha^−1^ had the highest soil moisture across the seasons and crop growth stages.

**Table 2:** Soil moisture (mm) at 0–40 cm soil depth at different crop growth stages under soil fertility and, soil & water conservation practices in the drylands of Tharaka-Nithi County, Kenya.

Source of variation		Days after planting and specific growth stages								
		SR 19			LR 20			SR 20		
SWC		**VP**	**FP**	**GFP**	**VP**	**FP**	**GFP**	**VP**	**FP**	**GFP**
**Conventional till**		78.21^a^	74.63^a^	74.83^a^	48.92^b^	49.16^b^	43.12^c^	43.61^c^	81.57^a^	37.08^a^
**Minimum till + mulch**		74.60^a^	72.25^a^	71.99^a^	55.10^ab^	55.60^a^	66.63^a^	64.87^a^	84.25^a^	35.32^a^
**Tied ridge**		76.58^a^	76.24^a^	75.40^a^	60.41^a^	55.12^a^	55.81^b^	59.02^b^	80.18^a^	38.08^a^
**Hsd**		2.418	1.585	4.014	6.402	5.041	8.796	5.821	4.846	2.584
**p value**		0.1041	0.4521	0.0628	<0.0001	0.0231	<0.0001	<0.0001	0.4301	0.1205
ISFM										
**120 N kg ha**^**−**^**^1^**		73.17^a^	71.22^b^	71.23^a^	47.42^b^	48.36^b^	42.42^bc^	42.61^c^	80.57^a^	36.28^a^
**60 N kg ha**^**−**^**^1^**		74.18^a^	73.83^b^	74.99^a^	54.15^a^	54.52^a^	54.41^b^	58.32^b^	83.15^a^	34.22^a^
**30 N kg ha**^**−**^**^1^**		76.58^a^	75.20^a^	74.40^a^	59.31^a^	54.62^a^	65.33^a^	63.17^a^	79.28^a^	37.00^a^
**Hsd**		3.418	3.205	3.184	6.472	9.061	13.764	11.258	14.246	3.485
**p value**		0.1164	0.0021	0.0544	<0.0001	<0.0001	<0.0001	<0.0001	0.0931	0.0525
SWC* ISFM										
Conventional Till*120 N kg ha^**−**^**^1^**	76.20		74.23	74.53	48.17	48.76	42.77	43.11	81.07	36.68
Conventional Till *60 N kg ha^**−**^**^1^**	75.69		72.925	73.41	51.535	51.84	54.225	53.39	82.36	35.65
Conventional Till*30 N kg ha-1	77.40		74.915	74.615	54.115	51.89	48.765	50.965	80.425	37.04
**Minimum Till** + mulch*120 kg ha^**−**^**^1^**	74.39		73.04	73.11	51.26	51.98	54.53	53.74	82.41	35.80
**Minimum Till** + mulch*60 kg ha^**−**^**^1^**	73.89		71.74	71.99	54.63	55.06	65.98	64.02	83.70	34.77
**Minimum Till** + mulch* 30 kg ha^**−**^**^1^**	75.59		73.73	73.20	57.21	55.11	60.52	61.60	81.77	36.16
Tied ridge* 120 N kg ha^**−**^**^1^**	75.38		75.04	74.82	53.92	51.74	49.12	50.82	80.38	37.18
Tied ridge* 60 N kg ha^**−**^**^1^**	74.88		73.73	73.70	57.28	54.82	60.57	61.10	81.67	36.15
Tied ridge * 30 N kg ha^**−**^**^1^**	76.58		75.72	74.90	59.86	54.87	55.11	58.67	79.73	37.54
**p value**	0.0160		0.0021	0.0404	<0.0001	<0.0001	<0.0001	<0.0001	0.0031	0.0305

SWC=Soil and water conservation; ISFM=Integrated soil fertility management; Till = Tillage; VP=Vegetative phase (0–45 days); FP=Floral phase (45–70 days); GFP = Grain filling phase (70–100 days). Means with the same superscript letter within the same column are not significantly different, separation done by turkey’s honestly significant difference at 95% level of confidence.

Manure plus fertilizer under minimum tillage with residue mulch increased soil moisture. This is because the minimum tillage with mulch reduced direct evaporation and runoff water losses [14]. In addition, manure and mineral fertilizer boosted the canopy cover through improved crop growth and foliation which contributed to reduced evaporation and runoff water losses [33]. Our results are consistent with the observation by Arora et al. [34], where direct evaporation and surface runoff water losses were reduced as a result of reduced temperature and improved infiltration rate under crop residue mulch. Similarly, Abdullah [35] observed minimum tillage with organic resources to have enhanced soil moisture more than conventional tillage under a long-term study due to reduced water loss.

Manure plus mineral fertilizer under conventional tillage could have enhanced soil moisture during the trial period as a result of hastened integration of manure into the soil by the tillage [36]. Manure binds micro-aggregates into macroaggregates particles, thus improving soil hydrological properties rate [37]. The same observation was made by Mutuku et al. [6], where soil organic inputs including manure under conventional tillage with crop residue mulch improved soil moisture. Treatments under tied ridges also enhanced soil moisture. This could have been due to the reduction of water loss through surface runoff and direct evaporation [38]. Tied ridges reduced the runoff water velocity, and retain water in the ditches, availing it for use by the crops [18]. The retaining of water in the ditches reduces the surface area of the water exposed for direct evaporation, hence reducing evaporation losses [18]. Kiboi et al. [38] made a similar observation where tied ridges reduced soil water loss through surface runoff and evaporation, improving soil moisture profile.

Soil moisture content is reduced with an increase in the rate of fertilization. The relatively low soil moisture under 120 N kg ha^−1^ rate compared to the 60 N kg ha^−1^ and 30 N kg ha^−1^ could be because of the high soil moisture consumption rate by the crops at a high fertilization rate, which resulted in the observed high yields [39]. High fertilization hastens crop growth and improves foliation, which translates to a high rate of soil moisture consumption [9]. This supports the argument by Steduto et al. [39], that increased crop transpiration accelerates soil moisture diminution rate. Oduor et al. [3], also observed faster soil moisture depletion under mineral fertilizer treatment, and attributed the observation to improved crop growth that accelerated soil moisture utilization.

The control steadily recorded low soil moisture in comparison with other treatments. This could be attributed to the continuous turning of the soil that exposed the top soils to evaporation losses under conventional tillage, especially with inadequate soil organic soil amendment mechanisms [36]. The application of 30 N kg ha^−1^ which is commonly applied by the farmers was not sufficient to supply the required nutrients to the crop. This prompted the relatively low rate of crop development, hence low foliation [25]. The inadequate application of fertilization rate and non-use of soil water conservation measures has been observed to reduce the yield and soil moisture as per various studies [[40], [41], [42]].

### 3.3 Treatment effect on soil water use efficiency

Treatment influenced soil WUE significantly for both total above-ground biomass and sorghum yield across the three seasons (Table 3). Above-ground biomass and sorghum grain yield WUE were significantly improved by 11–150% by minimum tillage with mulch, whereas it was enhanced by 50–65% by the tied ridges compared to the conventional tillage. Likewise, integrated soil fertility management significantly enhanced total above-ground biomass and sorghum grain yield during the trial period. Compared with 30 N kg ha^−1^, 120 N kg ha^−1^ enhanced the WUE by 25–66%, whereas 60 kg ha^−1^ enhanced the by 18–25%. Generally, minimum tillage with mulch under 120 N kg ha^−1^ of manure plus fertilizer had the highest interaction on WUE across the seasons. Hence, the best treatment combination for enhancing water use efficiency.

**Table 3:** Soil water use efficiency (kg m^−3^) of sorghum under soil fertility and, soil & water conservation practices in the drylands of Tharaka-Nithi County, Kenya.

Source of variation	WUE (Above ground biomass)			WUE (Grain yield)		
	**SR 2019**	**LR 2020**	**SR 2020**	**SR 2019**	**LR 2020**	**SR 2020**
SWC						
**Conventional tillage**	17.73^b^	4.79^c^	16.32^bc^	3.49^b^	1.38^c^	4.88^b^
**Minimum tillage + mulch**	19.51^a^	10.16^a^	23.30^a^	3.70^a^	4.04^a^	6.86^a^
**Tied ridge**	17.57^b^	8.23^ab^	18.42^b^	3.59^b^	2.25^b^	5.02^b^
**Hsd**	2.1336	3.569	3.895	0.092	0.858	0.981
**p value**	<0.0001	<0.0001	<0.0001	<0.0001	<0.0001	<0.0001
ISFM						
**120 N kg ha**^**−**^**^1^**	20.59^a^	6.44^b^	19.25^b^	3.65^a^	2.16^ab^	5.04^c^
**60 N kg ha**^**−**^**^1^**	16.39^b^	6.26^b^	21.96^a^	3.44^a^	2.39^ab^	6.79^a^
**30 N kg ha**^**−**^**^1^**	16.29^b^	8.45^a^	17.00^bc^	2.78^a^	2.87^a^	4.29^bc^
**Hsd**	3.486	2.069	2.595	0.092	0.697	0.981
**p value**	<0.0001	<0.0001	<0.0001	0.1074	0.0881	<0.0001
SWC*ISFM						
Conventional tillage*120 N kg ha^**−**^**^1^**	19.16	5.62	17.79	3.57	1.77	4.96
Conventional tillage *60 N kg ha^**−**^**^1^**	17.06	5.53	19.14	3.47	1.89	5.84
Conventional tillage *30 N kg ha-1	17.01	6.62	16.66	3.14	2.13	4.59
Minimum tillage + mulch*120 N kg ha^**−**^**^1^**	20.05	8.30	21.28	3.68	3.10	5.95
Minimum tillage + mulch* 60 N kg ha^**−**^**^1^**	17.95	8.21	22.63	3.57	3.22	6.83
Minimum tillage + mulch* 30 N kg ha^**−**^**^1^**	17.90	9.31	20.15	3.24	3.46	5.58
Tied ridges* 120 N kg ha^**−**^**^1^**	19.08	7.34	18.84	3.62	2.21	5.03
Tied ridges* 60 N kg ha^**−**^**^1^**	16.98	7.25	20.19	3.52	2.32	5.91
Tied ridges * 30 N kg ha^**−**^^1^Tied ridges* 30 N kg ha^**−**^**^1^**	16.98	7.25	20.19	3.52	2.32	5.91
**p value**	<0.0001	<0.0001	<0.0001	<0.0001	<0.0001	<0.0001

WUE=Water use efficiency; SWC=Soil and water conservation; ISFM=Integrated soil fertility management. Means with the same superscript letter within the same column are not significantly different, separation done by turkey’s honestly significant difference at 95% level of confidence.

The observed increase in WUE under animal manure could be due to the optimal utilization of the available water for crop production [43]. Like other organic resources, animal manure boosts soil physical properties and hence reduces soil moisture loss [44]. This improves soil hydrological properties and water use efficiency. Treatments with mineral fertilizer also improved water use efficiency. Mineral fertilizer improved grain yield and above-ground biomass by readily supplying the N required for the growth and development of crops [21]. The improved foliation enhances soil cover which reduces evaporation water loss leading to the observed improvement in water conservation [45]. Besides water conservation, fertilizer upon supply of crop nutrients contributed to improved yield, thus the high WUE [41]. Animal manure plus fertilizer enhanced the efficient utilization of water since animal manure simultaneously supplies nutrients to the crops, and contributes to reduced water loss by providing an organic amendment to the soil [2]. The organic effect helped in improving soil physical properties as manure acted as the binding agent. Furthermore, the integrated effect of animal manure and mineral fertilizer had a synergistic effect that improved the efficiency of the nutrient use, hence higher crop yield than when either is used in isolation [25]. Therefore, the highest gain in WUE was under the treatment that had both mineral fertilizer and manure. Molden et al. [11] and Adeboye et al. [41] made the same observation, whereby WUE was improved when the combination of organic and mineral fertilizers was used.

Treatment under conventional tillage, tied ridges, and minimum tillage with mulch enhanced water use efficiency. Under conventional tillage, the observed improvement in WUE could be because of accelerated nutrients released from manure due to the constant turning of the soil by tillage which speeds up the decomposition rate [46]. This ensures the effect of animal manure was apparent even over a short period [36]. Oicha et al. [47] made the same observation, where conventional tillage performed better than the minimum in terms of crop yield, and consequently water use efficiency. Tied ridges contributed to improved WUE by conserving soil water lost by erosion and direct evaporation [18]. Thus, improving water content in the soil profile available for crop utilization [48]. As a result, even a little amount of water in the soil was used optimally for crop production, hence the high WUE [17]. Minimum tillage with mulch on the other hand improved WUE by minimizing losses through direct evaporation and runoff losses [12]. The mulch reduced the velocity of surface runoff giving water more time to infiltrate into the soil for crop utilization [36]. Araya and Stroosnijder [18] also reported minimum tillage to have conserved soil moisture more than conventional tillage.

## 4 Conclusion

Minimum tillage with mulch was the best soil and water conservation practice for enhancing soil moisture, and hence WUE during the experimental period compared to other soil and water conservation practices. The fertilization rate of 30 N kg ha^−1^ had the highest soil moisture, whereas 120 N kg ha^−1^ had the highest water use efficiency. The interaction of manure plus fertilizer at 30 N kg ha^−1^ with minimum tillage with mulch, was the best in soil moisture conservation, whereas manure plus fertilizer at 120 N kg ha^−1^ with minimum tillage with mulch had the highest WUE. The high yield and WUE are because of the ability of the treatment combination to boost crop yield and conserve soil water, simultaneously. Therefore, the use of minimum tillage with mulch in combination with manure plus fertilizer at 120 N kg ha^−1^ should be encouraged to enhance sustainable agricultural productivity.

## Author contribution statement

Nathan Okoth oduor: Conceived and designed the experiments; Performed the experiments; Analyzed and interpreted the data; Wrote the paper.

Monicah Mucheru-Muna, Jayne N. Mugwe, Isaya Sijali and Nyabuga George: Conceived and designed the experiments; Analyzed and interpreted the data; Wrote the paper.

Daniel N. Mugendi: Conceived and designed the experiments; Contributed reagents, materials, analysis tools, or data; Wrote the paper.

## Data availability statement

Data will be made available on request.

## Declaration of interest’s statement

The authors declare no conflict of interest.

## Declaration of competing interest

The authors declare that they have no known competing financial interests or personal relationships that could have appeared to influence the work reported in this paper.
